# Both K63 and K48 ubiquitin linkages signal lysosomal degradation of the LDL receptor

**DOI:** 10.1194/jlr.M035774

**Published:** 2013-05

**Authors:** Li Zhang, Ming Xu, Elena Scotti, Zhijian J. Chen, Peter Tontonoz

**Affiliations:** * Howard Hughes Medical Institute and Department of Pathology and Laboratory Medicine, David Geffen School of Medicine, University of California at Los Angeles, Los Angeles, CA 90095; † ^3^Department of Molecular Biology, University of Texas Southwestern Medical Center, Dallas, TX 75390; § Howard Hughes Medical Institute, University of Texas Southwestern Medical Center, Dallas, TX 75390

**Keywords:** low density lipoprotein receptor, lysosomal protein degradation, proteasomal protein degradation, E3 ubiquitin ligase

## Abstract

Linkage-specific ubiquitination often leads to distinct cellular events. It has been difficult to establish definitively the requirement for a particular linkage in mammalian degradation pathways due to the inability to deplete endogenous ubiquitin while maintaining cell viability. The E3 ubiquitin ligase inducible degrader of the LDL receptor (IDOL) targets the low density lipoprotein receptor (LDLR) for degradation. The nature of the linkages employed to signal lysosomal degradation of the LDLR, and to signal proteasomal autodegradation of IDOL, have not been determined. We used an inducible RNAi strategy to replace endogenous ubiquitin with mutants lacking K48 or K63. We found that IDOL catalyzes the transfer of ubiquitin chains to itself and to the LDLR that do not contain exclusively K48 or K63 linkages. Thus, LDLR can be targeted to the lysosome by either K48 or K63 linkages. We further demonstrate that although both ubiquitin conjugating enzyme E2 (UBE2)Ds and UBE2N/V1 can catalyze LDLR ubiquitination in a cell-free system, UBE2Ds appear to be the major E2 enzymes employed by IDOL in cells, consistent with their ability to catalyze both K48 and K63 linkages. The results reveal mechanistic insight into the posttranscriptional control of lipoprotein uptake and provide a test of the requirement of linkage-specific ubiquitination for specific lysosomal and proteasomal degradation pathways in mammalian cells.

Ubiquitination is one of the most universal posttranslational protein modifications occurring in the cell (1). Ubiquitin, an evolutionarily highly conserved 76 amino acid polypeptide, is covalently attached to lysine residues of target proteins through a highly organized and hierarchical group of enzymes (2). Ubiquitin activating enzyme (E1) activates ubiquitin, forming a high-energy thioester bond between the C terminus of ubiquitin and E1. The activated ubiquitin is then transferred to a ubiquitin conjugating enzyme (E2). Following that, a large group of ubiquitin ligases (E3s) facilitate the transfer of ubiquitin from E2 to the lysine residues on the substrate proteins, either directly, or by forming an E3-ubiquitin intermediate (3). As there are seven lysine residues on ubiquitin, it is possible that during ubiquitination, new ubiquitin molecules could be added to one of the seven lysine residues of the previously conjugated ubiquitin (4). This process, called polyubiquitination, is a common cellular signal.

Depending on the usage of the lysine residues on ubiquitin, various linkage-specific ubiquitinations could happen. K48 and K63 linkage-specific ubiquitinations are the most predominant forms of polyubiquitination, accounting for 52 and 38% of all ubiquitination events respectively, in HEK293 cells (5). Because ubiquitination via different lysine linkages would result in distinct conformations of ubiquitin and thereby restrict the accessible lysines (6, 7), alternate linkage in polyubiquitination is relatively rare, but has nonetheless been reported (8–10).

In recent years, the role of different linkage-specific polyubiquitinations has begun to be elucidated. A K48-linked polyubiquitin chain has been shown to be sufficient to target a model substrate to the 26S proteasome, and has been proved to be a principal proteasome delivery signal for multiple short-lived proteins in the cell (11, 12). In contrast to the proteolytic role of the K48-specific linkage, the K63-specific linkage has been demonstrated to regulate a variety of nonproteolytic cellular functions, including DNA damage repair (13), stress responses (14), and inflammatory pathways (15). Importantly, K63-specific ubiquitination has also been shown to facilitate the endocytosis of membrane proteins (16). In yeast strains in which the endogenous ubiquitin was depleted either by eliminating all ubiquitin-coding genes (12, 13), or by mutating the genes essential for free ubiquitin recycling (17), definitive evidence has been obtained that K63-specific polyubiquitination is required for the endocytosis, the vacuole sorting, and the degradation of membrane proteins such as uracil permease (18), Gap1p permease (19), and carboxypeptidase S (20). Studies in mammalian cells suggest that K63-specific polyubiquitination is involved in various stages of the internalization, the lysosome sorting, and the degradation processes of membrane proteins, including the epidermal growth factor receptor (EGFR) (21, 22), nerve growth factor receptor TrkA (23), major histocompatibility complex class I molecules (24), and the prolactin receptor (25). However, due to the technical difficulty in eliminating endogenous ubiquitin genes in mammalian cells, direct evidence is still lacking whether the K63-specific ubiquitin linkage is an indispensible element in the endocytosis and lysosomal degradation of membrane proteins in mammalian cells.

We have demonstrated that under conditions of elevated intracellular cholesterol, the liver X receptor (LXR) induces the transcription of the E3 ubiquitin ligase inducible degrader of the LDL receptor (IDOL), which ubiquitinates and facilitates the degradation of the low-density lipoprotein receptor (LDLR) (26–28). Typical of an E3 ubiquitin ligase, IDOL also ubiquitinates itself and thereby promotes its own turnover. Interestingly, autodegradation of IDOL appears to occur through the proteasomal pathway, whereas IDOL-dependent degradation of the LDLR occurs via the lysosome (29). Indirect evidence has suggested that K63-specific ubiquitination could be involved in the degradation of the LDLR (30). However, it has not been rigorously tested whether K48 or K63 linkage-specific ubiquitination is actually required for the ubiquitination and degradation of IDOL or the LDLR.

For most genes it is relatively straightforward to generate a stable knockdown cell and then replace a mutant version of the gene of interest with an expression vector. But there are four ubiquitin genes in mammals, and knocking them all down kills the cell. Thus, standard small inhibitory RNA (siRNA) knockdown studies are impractical. The only way to introduce mutant ubiquitins into a null background is to knockdown all 4 ubiquitin genes while simultaneously introducing the expression of a new ubiquitin molecule to keep the cells alive. Recently, a ubiquitin replacement strategy was developed in which a tetracycline-inducible RNA inhibition (RNAi) was used to replace the endogenous ubiquitin proteins with ubiquitin mutants. This strategy was previously employed to determine the requirement for K63-specific ubiquitin chains in IκB kinase (IKK) activation by IL-1β (31).

Here we have employed the ubiquitin replacement strategy of Xu et al. (31) to determine the nature of the ubiquitin linkages involved in IDOL-dependent protein degradation. We initially hypothesized that IDOL might employ K48-polyubiquitin chains to target itself for proteasomal degradation, and K63-specific linkages to trigger lysosomal degradation of the LDLR. Contrary to expectations, however, the degradation of neither IDOL nor the LDLR was exclusively mediated by K48- or K63-specific ubiquitination, strongly suggesting that either linkage can signal proteasomal and lysosomal degradation. We also found that ubiquitin conjugating enzyme E2 (UBE2)N/V1, a heterodimeric ubiquitin E2 enzyme that specifically catalyzes K63-specific ubiquitin linkage, is not required for the ubiquitination and degradation of the LDLR. This study provides a test of the requirement of linkage-specific ubiquitination for lysosomal and proteasomal protein degradation pathways in mammalian cells.

## MATERIALS AND METHODS

### Reagents

The synthetic LXR ligand GW3965 was provided by T. Wilson (GlaxoSmithKline). MG132, tetracycline, bafilomycin, and mevalonic acid were purchased from Sigma-Aldrich. Simvastatin sodium salt was purchased from Calbiochem.

### Plasmids and constructs

The pSA2-N-TAP plasmid that contains the 3xFLAG-Strep tag and the pcDNA-V5-DEST plasmid were kind gifts from Dr. E. Saez (Scripps Institute). pDONR221, pET300N-DEST, and pcDNA-DEST47 plasmids were purchased from Invitrogen. The DNA sequence of the human Idol gene was amplified from a pcDNA-V5::hIdol construct as previously reported (28), and was then subcloned into pSA2-N-TAP plasmid. The IDOL C387A mutation for the pSA2-N-TAP::hIdol constructs was introduced by site-directed mutagenesis. The DNA sequence of the human Ldlr gene was amplified from pCB6-hLdlr (a kind gift from Dr. K. Matter, University College London, UK) with a predesigned primer encoding a V5 tag to the C terminus of the coding sequence of hLdlr. The DNA sequence of the tagged hLdlr was then subcloned into pcDNA-DEST47 using the Gateway technology (Invitrogen). For the GFP-tagged hLDLR, hLdlr was amplified with the stop codon removed and was then subcloned into pcDNA-DEST47 using the Gateway technology. pcDNA3.1-(HA-Ub)_6_ was a kind gift from Dr. J. Wohlschlegel (University of California at Los Angeles). The K48R and K63R mutations for the pcDNA3.1-(HA-Ub)_6_ construct were introduced by site-directed mutagenesis. The human E2 genes hUbe2d2, hUbe2n, and hUbe2v1 were cloned from HEK293T cell cDNA and were then sequentially subcloned into pDONR221 and pET300N-DEST using the Gateway technology for *Escherichia coli* protein expression. In addition, the hUbe2d2 and hUbe2n genes in the pDONR221::hUbe2d2 and pDONR221::hUbe2n constructs were subcloned into pcDNA-V5-DEST plasmid using the Gateway technology. The UBE2D2 C85A and the UBE2N C87A mutations for the pcDNA-V5::hUbe2d2 and the pcDNA-V5::hUbe2n constructs, respectively, were introduced by site-directed mutagenesis.

### Antibodies

Rabbit anti-hLDLR antibody was purchased from Cayman Chemicals. Rabbit anti-actin and mouse anti-FLAG M2 antibodies were purchased from Sigma-Aldrich. Mouse anti-V5 antibody, HRP-conjugated goat anti-mouse IgG, and goat anti-rabbit IgG were purchased from Invitrogen. Rabbit anti-V5 antibody was purchased from Abcam. Rabbit anti-GFP antibody was purchased from Clontech. Mouse anti-HA antibody was purchased from Covance. All commercially available antibodies were used according to the manufacturers’ instructions.

### Cell culture and transfection

HEK293T cells and the engineered U2OS cells for ubiquitin replacement were maintained in DMEM (Invitrogen) supplemented with 10% fetal bovine serum (Omega), 2 mM l-glutamine (Invitrogen), 50 U/ml penicillin (Invitrogen), and 50 µg/ml streptomycin (Invitrogen). Cells were grown in a humidified incubator at 37°C and 5% CO_2_ atmosphere. HEK293T cells were transfected using FuGENE 6 reagents (Roche) according to the manufacturer's instructions.

### Generation and amplification of adenoviral particles

Ad-mIdol particles were generated as previously described (28). For the Ad-hLdlr-V5 particle, the DNA sequence of hLdlr was amplified from pCB6-hLdlr with the stop codon removed. The hLdlr sequence was then subcloned sequentially into pDONR221 and pAd-CMV-V5-DEST (Invitrogen) with the Gateway technology. Viruses were amplified, purified, and titered by Viraquest.

### In vitro ubiquitination assay

The in vitro IDOL autoubiquitination assay and LDLR ubiquitination assay were carried out as previously described (29).

### Immunoblotting

Proteins were resolved on 4–12% gradient SDS-PAGE (Invitrogen) using standard protocols. The protein was electrophoretically transferred to nitrocellulose membranes (Amersham Biosciences) and blocked with milk solution (150 mM NaCl, 20 mM Tris, 5% milk, 0.2% Tween, pH 7.5) to quench nonspecific protein binding. The blocked membranes were probed sequentially with primary and secondary antibodies diluted in the milk solution, and the bands were visualized with the ECL kit (Amersham Biosciences).

## RESULTS

### IDOL and LDLR ubiquitination does not exclusively depend on K48- or K63-linked ubiquitin

**Fig. 1.**
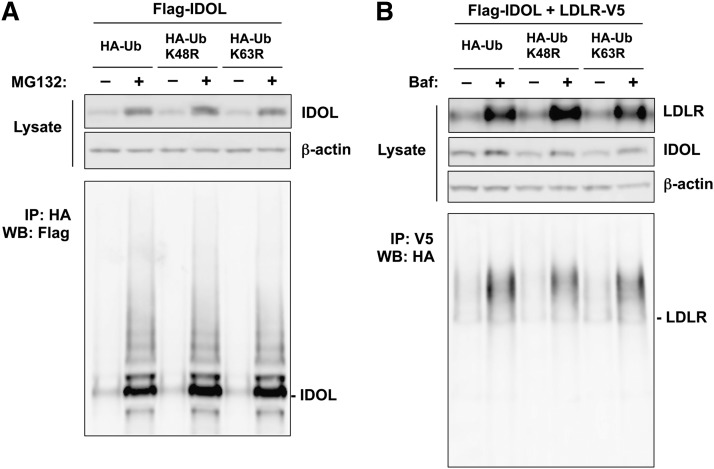
The ubiquitination of IDOL and the LDLR does not exclusively depend on K48- or K63-linked ubiquitin in an overexpression system. A: HEK293T cells were cotransfected with FLAG-tagged IDOL and HA-tagged wild-type K48R or K63R ubiquitin. Cells were treated with 25 μM MG132 for 4 h before being lysed in RIPA buffer. Proteins in the lysates were analyzed by immunoprecipitation and immunoblotting. B: HEK293T cells were cotransfected with FLAG-tagged IDOL, V5-tagged LDLR, and HA-tagged wild-type K48R or K63R ubiquitin. Cells were treated with 50 nM bafilomycin (Baf) for 4 h before being lysed in RIPA buffer. Proteins in the lysates were analyzed by immunoprecipitation and immunoblotting. IP, immunoprecipitation; WB, Western blotting.

To examine whether the ubiquitination of IDOL is mediated exclusively by K48- or K63-linked ubiquitin, we transfected HEK293T cells with FLAG-tagged IDOL, together with either HA-tagged wild-type ubiquitin, or ubiquitin mutants harboring lysine to arginine mutations at the K48 or K63 residues. We hypothesized that if the ubiquitination of IDOL was exclusively mediated by K48 or K63 linkage, mutations on these residues would prevent the HA-tagged mutant ubiquitin from participating in the elongation of polyubiquitin chains, and therefore would reduce the ubiquitination revealed by the HA tag. As we previously reported, IDOL undergoes active autoubiquitination and autodegradation (29). Therefore, in order to better demonstrate the ubiquitination of IDOL, we treated these transfected cells with proteasome inhibitor MG132 prior to harvest. As expected, this treatment led to the accumulation of IDOL protein (**Fig. 1A**). We did not observe any difference in IDOL ubiquitination between cells transfected with wild-type ubiquitin and cells transfected with either K48R or K63R mutant ubiquitin, as revealed by the HA tag (Fig. 1A). This result suggested that IDOL ubiquitination does not depend exclusively on the K48 or K63 linkage.

We also examined whether the ubiquitination of the LDLR was mediated exclusively by K48- or K63-specific linkages in this system. Because the degradation of the LDLR is lysosome dependent, we treated transfected cells prior to harvest with bafilomycin to preserve ubiquitinated LDLR. This treatment resulted in the accumulation of the LDLR but had little effect on IDOL (Fig. 1B). As revealed by the HA tag, there was no discernible difference in LDLR ubiquitination between cells transfected with wild-type ubiquitin and cells transfected with either K48R or K63R mutant ubiquitin (Fig. 1B). This result suggested that, similar to IDOL autoubiquitination, the ubiquitination of the LDLR does not exclusively depend on K48- or K63-linked ubiquitin.

**Fig. 2.**
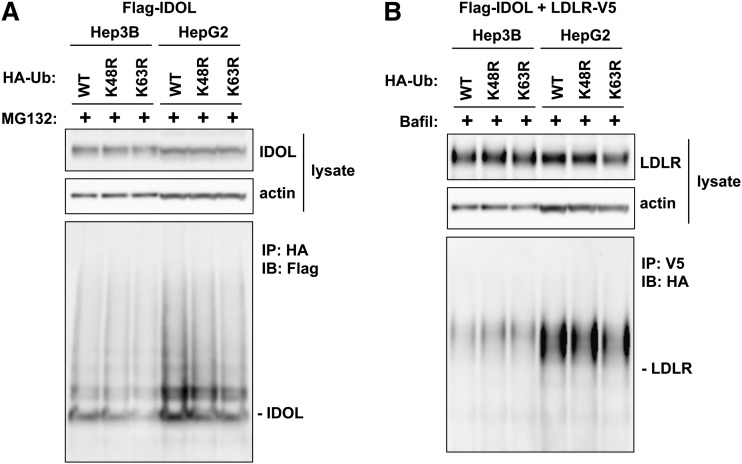
The ubiquitination of IDOL and the LDLR does not exclusively depend on K48- or K63-linked ubiquitin in hepatocyte cell lines. A: Hep3B and HepG2 cells were cotransfected with FLAG-tagged IDOL and HA-tagged wild-type K48R or K63R ubiquitin. Cells were treated with 25 μM MG132 for 4 h before lysis in RIPA buffer. Proteins in the lysates were analyzed by immunoprecipitation and immunoblotting. B: Hep3B and HepG2 cells were cotransfected with FLAG-tagged IDOL, V5-tagged LDLR, and HA-tagged wild-type K48R or K63R ubiquitin. Cells were treated with 50 nM bafilomycin (Bafil) for 4 h before lysis in RIPA buffer. Proteins in the lysates were analyzed by immunoprecipitation and immunoblotting. IP, immunoprecipitation; WB, Western blotting.

Because the liver is an important organ contributing to cholesterol homeostasis, we next sought to investigate lysine-specific ubiquitination of IDOL and the LDLR in two different hepatocyte cell lines, Hep3B and HepG2. We transfected these two cell lines with IDOL and/or the LDLR, together with HA-tagged wild-type ubiquitin, or HA-tagged ubiquitin mutants harboring lysine to arginine mutations at the K48 or K63 residues. In both hepatocyte cell lines we readily observed the formation of polyubiquitin chains from the transfected wild-type, K48R, and K63R ubiquitin on IDOL (**Fig. 2A**) and the LDLR (Fig. 2B). The levels of IDOL and LDLR ubiquitination observed were similar between cells transfected with wild-type ubiquitin and cells transfected with either K48R or K63R mutant ubiquitin. These results suggest that the ubiquitination of IDOL and the LDLR does not exclusively depend on K48- or K63-linked ubiquitin in hepatocytes.

### Characterization of cells in which endogenous ubiquitin is replaced

**Fig. 3.**
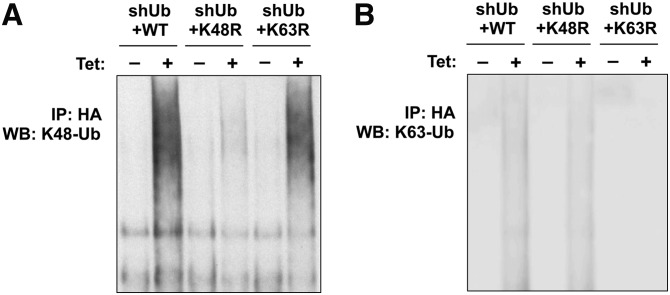
Characterization of ubiquitin-replaced U2OS cells. Stably transfected U2OS cells (shUb+WT, shUb+K48R, and shUb+K63R) were cultured in the absence (−) or presence (+) of 1 μg/ml tetracycline (Tet) for 72 h before lysis in RIPA buffer. Proteins in the lysates were immunoprecipitated with HA antibody and immunoblotted with antibodies specifically recognizing (A) K48-linked ubiquitin and (B) K63-linked ubiquitin. IP, immunoprecipitation; WB, Western blotting.

To further investigate whether the ubiquitination and the degradation of IDOL and the LDLR are exclusively dependent on K48- or K63-specific ubiquitin linkage, we made use of a previously described inducible ubiquitin replacement system (31). In this system, endogenous ubiquitin in stable U2OS cell lines is inducibly eliminated by shRNA (shUb) and replaced by exogenously expressed HA-tagged wild-type ubiquitin (shUb+WT) or ubiquitin mutants harboring point mutations on the K48 (shUb+K48R) or K63 residues (shUb+K63R). Both the shRNA and the replacement ubiquitin are under the control of a tetracycline-activated promoter. This system has been successfully utilized to differentiate the distinct ubiquitin linkages involved in IKK activation induced by tumor necrosis factor-α versus IL-1β (31). We initially set out to validate the efficacy of the system using antibodies recognizing K48- or K63-specific ubiquitination. After induction with tetracycline for 48 h, we found that although the formation of K48-specific ubiquitin linkages was intact in shUb+WT cells and shUb+K63R cells, it was markedly inhibited in shUb+K48R cells (**Fig. 3A**). Similarly, the formation of K63-specific ubiquitin linkages was inhibited in shUb+K63R cells, but not in shUb+WT cells or shUb+K48R cells (Fig. 3B). In addition, consistent with previous work (31), shUb cells and shUb+K48R cells were viable for only 72 h, likely because the lack of K48-specific ubiquitination is detrimental to vital cellular functions. In contrast, shUb+K63R cells were viable for 120 h, and shUb+WT cells are viable for a much longer period (data not shown).

### K48- and K63-specific ubiquitin linkages are dispensable in IDOL autoubiquitination and autodegradation

**Fig. 4.**
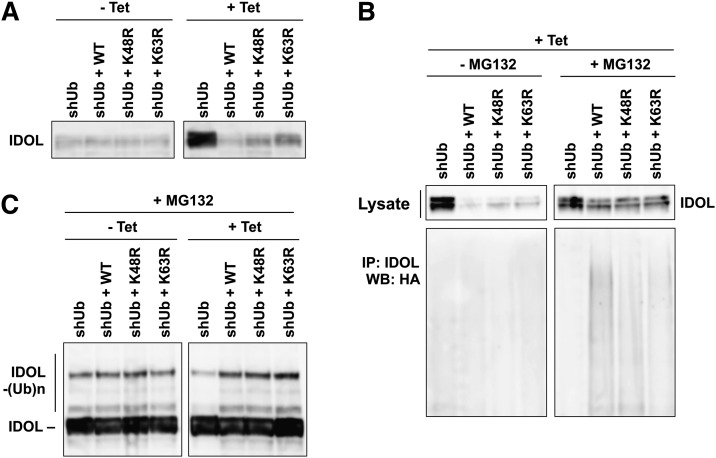
K48- and K63-specific ubiquitin linkages are dispensable for IDOL autoubiquitination and autodegradation. A: U2OS cell lines were cultured in the absence (−) or presence (+) of 1 μg/ml tetracycline (Tet) for 48 h before infection with adenovirus encoding IDOL. Cells were lysed in RIPA buffer for 24 h after the adenovirus infection. Proteins in the lysates were immunoblotted with an IDOL monoclonal antibody. B: U2OS cells were cultured in the presence of 1 μg/ml tetracycline for 48 h before infection with adenovirus encoding IDOL. Cells were lysed in RIPA buffer for 24 h after the adenovirus infection, with the last 4 h in the presence of 25 μM MG132. Proteins in the lysates were immunoprecipitated with an antibody-recognizing IDOL and immunoblotted with HA antibody. C: U2OS cells were cultured in the absence or presence of 1 μg/ml tetracycline for 48 h before being infected with adenovirus encoding IDOL. Cells were lysed in RIPA buffer for 24 h after adenovirus infection, with the last 4 h in the presence of 25 μM MG132. Proteins in the lysates were immunoblotted for IDOL. IP, immunoprecipitation; WB, Western blotting.

To explore the requirement for K48 and K63 ubiquitination in the autoubiquitination and autodegradation of IDOL using this replacement system, we cultured the four lines of engineered U2OS cells (shUb, shUb+WT, shUb+K48R, and shUb+K63R) in the absence or presence of tetracycline for 48 h, then infected them with adenovirus-encoding IDOL for 24 h. In all four cells lines grown in the absence of tetracycline, IDOL underwent active autodegradation and did not accumulate (**Fig. 4A**). However, in cells growing in the presence of tetracycline, where ubiquitin replacement took place, the autodegradation of IDOL was severely inhibited in shUb cells, as evidenced by the accumulation of IDOL. In contrast, we did not observe IDOL accumulation in shUb+WT, shUb+K48R, or shUb+K63R cells, indicating that the lack of the K48 or the K63 residues of ubiquitin did not prevent the autodegradation of IDOL (Fig. 4A).

Meanwhile, we also examined IDOL autoubiquitination in these cells. In order to reveal the ubiquitination of IDOL, we treated the cells with proteasome inhibitor MG132. With MG132 treatment, there was no difference in the amounts of IDOL observed in the four cell lines, because ubiquitinated IDOL could not be degraded under this circumstance (Fig. 4B). We observed that in shUb+WT cells, IDOL was polyubiquitinated as expected with the replaced wild-type ubiquitin (Fig. 4B). Importantly, IDOL was also polyubiquitinated in shUb+K48R and shUb+K63R cells with the replaced mutant ubiquitin (Fig. 4B), indicating that the K48 and the K63 residues of ubiquitin are not required for the assembly of polyubiquitin chains on IDOL.

Furthermore, we sought to compare the overall ubiquitination levels of IDOL before and after the endogenous ubiquitin was replaced. To this end, we treated U2OS cells growing in the absence or presence of tetracycline with MG132. Under these conditions, the autodegradation of IDOL was inhibited and ubiquitin conjugation to expressed IDOL could be directly revealed by an antibody against IDOL. We observed IDOL ubiquitination in all cell lines grown in the absence of tetracycline (Fig. 4C). In cells grown in the presence of tetracycline to replace endogenous ubiquitin, we still observed similar levels of IDOL ubiquitination in shUb+WT, shUb+K48R, and shUb+K63R cells (Fig. 4C). There was no IDOL ubiquitination in shUb cells grown in the presence of tetracycline, probably because the endogenous ubiquitin was depleted in these cells without any exogenous repletion (Fig. 4C). Because IDOL autodegradation was severely inhibited only in shUb cells, these results further confirm the functional connection between the ubiquitination and the degradation of IDOL. Taken together, these results indicate that the autoubiquitination and autodegradation of IDOL do not require the K48 or the K63 residues of ubiquitin, and therefore are not exclusively mediated by K48- or K63-linked ubiquitin chains.

### The ubiquitination and degradation of the LDLR do not require K48 or K63 ubiquitin

**Fig. 5.**
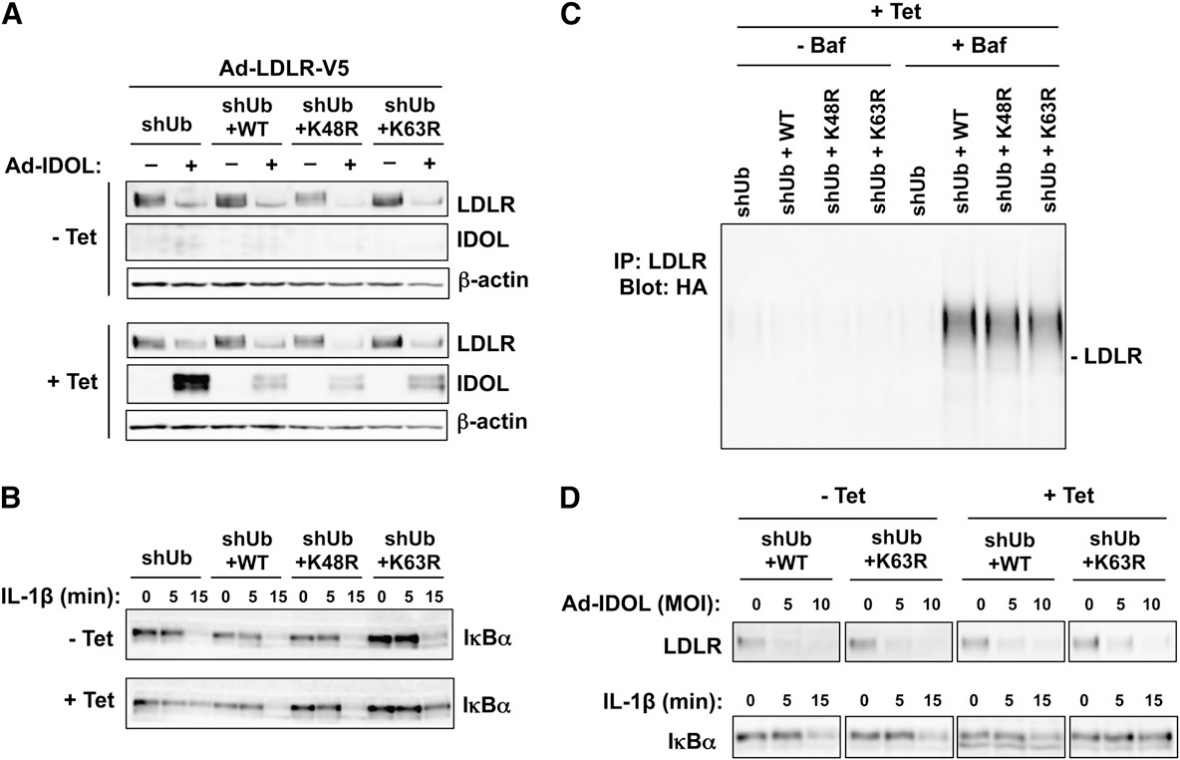
The ubiquitination and degradation of the LDLR do not require K48- or K63-specific ubiquitin linkages. A: U2OS cell lines were cultured in the absence (−) or presence (+) of 1 μg/ml tetracycline (Tet) for 48 h before being infected with adenoviral vectors encoding LDLR and/or IDOL as indicated. Cells were lysed in RIPA buffer for 24 h after adenovirus infection. Proteins in the lysates were immunoblotted with the indicated antibodies. B: U2OS cell lines were cultured in the absence or presence of 1 μg/ml tetracycline for 72 h and treated with 10 ng/ml IL-1β for the indicated times before being lysed in RIPA buffer. Proteins in the lysates were analyzed by immunoblotting. C: U2OS cells were cultured in the presence of 1 μg/ml tetracycline for 48 h before infections with adenoviral vectors encoding IDOL and V5-tagged LDLR. Cells were lysed in RIPA buffer for 24 h after the adenovirus infection, with the last 4 h in the presence of 50 nM bafilomycin (Baf). Proteins in the lysates were immunoprecipitated with an antibody recognizing LDLR and immunoblotted with HA antibody. D: Upper panel: U2OS cell lines were cultured in the absence or presence of 1 μg/ml tetracycline for 96 h before coinfection with adenovirus encoding the LDLR and the indicated titers of adenovirus encoding IDOL. Cells were lysed in RIPA buffer for 24 h after adenovirus infection. Proteins in the lysates were immunoblotted with the indicated antibody. Lower panel: U2OS cells were cultured in the absence or presence of 1 μg/ml tetracycline for 120 h and treated with 10 ng/ml IL-1β for the indicated times before lysis in RIPA buffer. Proteins in the lysates were immunoblotted with the indicated antibody.

Next, we sought to determine to role of K48- and K63-specific ubiquitin linkages in the ubiquitination and degradation of the LDLR. We cultured U2OS cells in the absence or presence of tetracycline for 48 h then coinfected them with adenoviruses respectively encoding IDOL and the LDLR for another 24 h. In all four cells lines of cells grown in the absence of tetracycline, the LDLR was efficiently degraded by IDOL (**Fig. 5A**). However, in cells grown in the presence of tetracycline, where the endogenous ubiquitin was replaced, the degradation of the LDLR was moderately inhibited in shUb cells, compared with that in the cells growing in the absence of tetracycline. We did not observe any difference in LDLR degradation in shUb+WT, shUb+K48R, or shUb+K63R cells between the conditions of absence versus presence of tetracycline (Fig. 5A), indicating that the lack of the K48 or the K63 residues of ubiquitin did not inhibit IDOL-mediated LDLR degradation. Therefore, we conclude that the degradation of the LDLR by IDOL does not require the K48 or K63 residues of ubiquitin.

In a parallel experiment, we treated U2OS cells grown in the absence or presence of tetracycline with IL-1β. This treatment leads to the phosphorylation and degradation of IκBα, a process previously established to be dependent on K63-specific ubiquitination (31). We found that in all four cell lines grown in the absence of tetracycline, IκBα was degraded as soon as 15 min after the addition of IL-1β (Fig. 5B). In the presence of tetracycline, however, IκBα was still degraded in shUb+WT and shUb+K48R cells, but degradation was markedly inhibited in shUb and shUb+K63R cells (Fig. 5B). This finding is consistent with the requirement for K63-linked ubiquitin chains in the degradation of IκBα, and validates the ability of this system to distinguish requirement for linkage-specific ubiquitination in biological processes.

We also investigated the role of K48 and K63 ubiquitin linkages in the polyubiquitination of the LDLR. As the degradation of LDLR occurs via the lysosomal pathway, in order to preserve the ubiquitinated LDLR, we used the lysosomal inhibitor bafilomycin to prevent ubiquitinated LDLR from being degraded. In the presence of tetracycline, the levels of polyubiquitination of the LDLR in shUb+K48R and shUb+K63R cells were comparable to that in shUb+WT cells (Fig. 5C). This result demonstrates that the K48 and the K63 residues of ubiquitin are not required for the assembly of polyubiquitin chains on the LDLR.

Several recent studies have demonstrated important roles of K63-specific ubiquitination in the internalization and degradation of certain membrane proteins (21–25). Therefore, we sought to further confirm whether the K63-specific linkage is required for the ubiquitination and the degradation of the LDLR with more stringent experiments. In contrast to shUb and shUb+K48R cells, which die within 72 h in the presence of tetracycline, shUb+WT and shUb+K63R cells survive for 120 h, allowing for a more thorough ubiquitin replacement. We cultured shUb+WT and shUb+K63R cells in the absence or presence of tetracycline for 96 h then coinfected them with adenoviruses encoding IDOL and the LDLR for another 24 h. We found that in shUb+WT and shUb+K63R cells growing in the presence of tetracycline, the LDLR was still efficiently degraded by IDOL (Fig. 5D). As expected, the degradation of IκBα induced by IL-1β was inhibited in shUb+K63R cells, but not in shUb+WT cells, in the presence of tetreacycline (Fig. 5D).

**Fig. 6.**
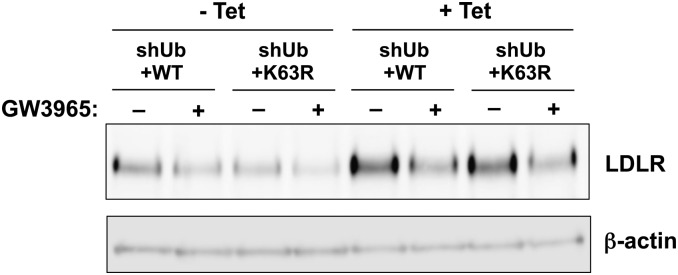
LDLR degradation induced by LXR activation does not require K48- or K63-specific ubiquitin linkage. U2OS cell lines were cultured in the absence (−) or presence (+) of 1 μg/ml tetracycline (Tet) for 108 h. In the absence or presence of tetracycline, cells were treated with 5 µM simvastatin and 100 µM mevalonic acid, with or without 1µM GW3965 for 12 h. Cells were lysed in RIPA buffer and proteins in the lysates were immunoblotted with the indicated antibodies.

We previously reported that the activation of LXR by the synthetic ligand GW3965 induces the expression of IDOL and consequently leads to the degradation of the LDLR. Therefore we sought to determine whether K63-specific ubiquitination was required in this context. We cultured shUb+WT and shUb+K63R cells in the absence or presence of tetracycline for 108 h then treated them with GW3965 for 12 h. Remarkably, GW3965 treatment induced the degradation of LDLR in both shUb+WT and shUb+K63R cells grown in the presence of tetracycline (**Fig. 6**). Taken together, these results further confirm that the degradation of LDLR does not require K63-specific ubiquitination.

### UBE2D rather than UBE2N/V1 is the primary biological partner for IDOL-mediated ubiquitination and degradation

**Fig. 7.**
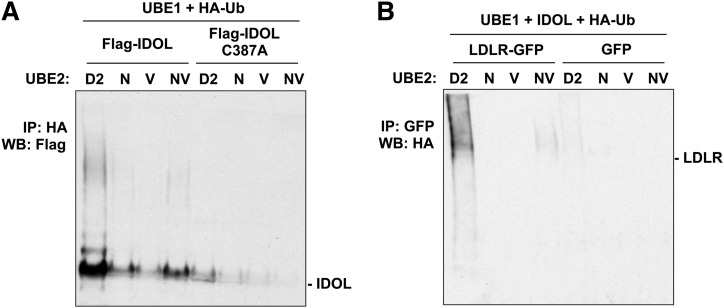
UBE2N/V1 does not efficiently couple with IDOL to ubiquitinate IDOL or the LDLR in vitro. A: Immunoprecipitated FLAG-tagged IDOL and IDOL C387A mutant were incubated with UBE1, HA-ubiquitin, and the indicated UBE2 proteins. IDOL ubiquitination was detected by immunoblotting for FLAG-tagged IDOL associated with HA-tagged ubiquitin. B: Membrane preparation of HEK293T cells expressing LDLR-GFP or GFP alone were incubated with UBE1, tandem affinity purified IDOL, HA-ubiquitin, and the indicated UBE2 proteins. LDLR was then immunoprecipitated with an anti-GFP antibody. The ubiquitination of LDLR was detected by immunoblotting for HA-tagged ubiquitin associated with the LDLR. IP, immunoprecipitation; WB, Western blotting.

In a previous study we demonstrated that ubiquitin E2 enzymes in the UBE2D family are capable of catalyzing IDOL-mediated ubiquitination and degradation of the LDLR (29). The UBE2N/V1 heterodimer mediates the assembly of K63-specific polyubiquitin chains (32, 33), which are believed to be involved in the internalization and degradation of multiple membrane proteins (21, 23–25). A recent report suggested that UBE2N/V1 may also be a biological partner for IDOL (30). Therefore, we sought to address whether IDOL-mediated ubiquitination and degradation of the LDLR could also be catalyzed by UBE2N/V1 in our system. We first examined in in vitro ubiquitination assays whether UBE2N/V1 could catalyze the autoubiquitination of IDOL and the ubiquitination of the LDLR. In the autoubiquitination assay, we mixed IDOL immunoprecipitated from HEK293T cells stably expressing FLAG-tagged IDOL, and crude lysates from *E. coli* expressing His-tagged UBE2 proteins, together with recombinant human UBE1, recombinant HA-tagged ubiquitin, and the ATP-generating system. To ensure the validity of the comparison, same amounts of UBE2 proteins from the same batch of preparation, as determined by Coomassie blue staining (data not shown), were used in the assay. IDOL ubiquitination was then revealed by ubiquitin immunoprecipitation and IDOL immunoblotting. Consistent with prior work (29), robust polyubiquitinated IDOL was detected in the presence of UBE2D2 (**Fig. 7A**). We found that UBE2N/V1 was also capable of catalyzing the polyubiquitination of IDOL in this assay, although the activity was substantially lower than that of UBE2D2 (Fig. 7A). As expected, all UBE2 proteins failed to ubiquitinate an IDOL mutant harboring a cysteine mutation in the catalytic RING domain (C387A) in this assay (Fig. 7A).

We also assayed LDLR ubiquitination by expressing an LDLR-GFP fusion or GFP control in HEK293T cells and preparing membrane fractions by permeabilizing the plasma membrane and removing cytosolic proteins (34). We mixed the membrane preparation with tandem affinity purified IDOL, crude lysates from *E. coli* expressing His-tagged UBE2 proteins, recombinant UBE1, recombinant HA-tagged ubiquitin, and the ATP-generating system. After the in vitro ubiquitination reaction, the membrane preparation was lysed and the LDLR was immunoprecipitated. The ubiquitination of the LDLR was then assayed by immunoblotting for ubiquitin. We found that a substantial amount of polyubiquitinated LDLR was formed in the presence of UBE2D2, whereas the formation of polyubiquitinated LDLR in the presence of the UBE2N/V1 heterodimer was barely detectable (Fig. 7B). In the presence of UBE2N or UBE2V1 alone, we could not detect any formation of polyubiquitinated LDLR (Fig. 6B).

**Fig. 8.**
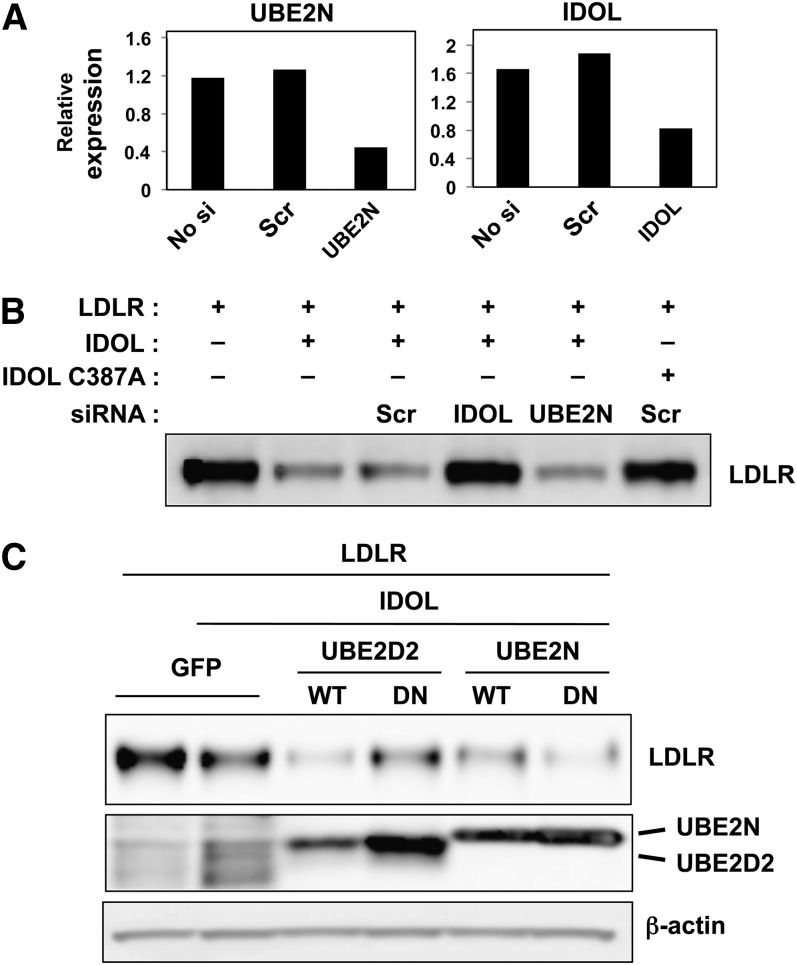
Inhibition of UBE2N/V1 expression and function does not prevent LDLR degradation. A: Quantitative PCR analysis of RNA samples collected from HEK293T cells tranfected with the indicated siRNAs. B: HEK293T cells were cotransfected with LDLR and IDOL, together with the indicated siRNAs. Cells were lysed in RIPA buffer and proteins in the lysates were immunoblotted with the indicated antibody. C: Immunoblot analysis of protein levels in HEK293T cells transfected with WT or dominant negative (DN) UBE2D2 or UBE2N, in addition to LDLR and IDOL.

We further investigated the role of UBE2N/V1 in the ubiquitination and degradation of the LDLR using the RNA interference technique in transfected HEK293T cells. With the transfection of an siRNA targeting UBE2N, we were able to reduce the mRNA of UBE2N by 70% (**Fig. 8A**) in these cells. As a control, transfection of siRNA targeting IDOL reduced IDOL mRNA by about 50% (Fig. 8A). In HEK293T cells cotransfected with IDOL and the LDLR, RNA interference of IDOL led to the inhibition of LDLR degradation, but RNA interference of UBE2N did not affect the degradation of the LDLR (Fig. 8B), suggesting that the ubiquitination and the degradation of the LDLR in these cells was not dependent on the function the UBE2N/V1 heterodimer.

We previously demonstrated that overexpression of the dominant negative version of UBE2D2 (C85A) (35), a cognate E2 enzyme for IDOL and the LDLR, could inhibit the degradation of the LDLR (29). In order to determine whether UBE2N/V1 was also a cognate E2 enzyme for IDOL and the LDLR, we examined whether the dominant negative version of UBE2N (C87A) could also inhibit LDLR degradation. Compared with its wild-type counterpart, overexpression of the dominant negative version of UBE2D2 markedly inhibited the degradation of the LDLR. However, no inhibitory effect was observed when the dominant negative version of UBE2N was overexpressed (Fig. 8C). Taken together, these results suggest that IDOL-mediated ubiquitination and degradation of the LDLR are catalyzed primarily by UBE2Ds rather than by the UBE2N/V1 heterodimer. This observation is consistent with the ability of UBE2D enzymes to catalyze the formation of both K48- and K63-linked ubiquitin chains.

## DISCUSSION

The abundant expression of endogenous ubiquitin in mammalian cells has made it extremely difficult to study the requirement for linkage-specific ubiquitination in protein degradation and other cellular functions. In this study, we have overcome this problem by using an inducible RNAi strategy that replaces endogenous ubiquitin with ubiquitin mutants lacking the K48 or the K63 residue (31). We determined that the ubiquitination and degradation of IDOL and the LDLR are not dependent on the presence of the ubiquitin K48 or K63 residues. Therefore, contrary to our original expectation given the substantial literature suggesting specific roles for K48 and K63 linkages in proteasomal and lysosomal degradation respectively, there is no requirement of either K48- or K63-specific ubiquitin chains for the ubiquitination and degradation of IDOL and the LDLR. We have also found that UBE2N/V1, a heterodimeric ubiquitin E2 enzyme that specifically catalyzes K63-specific ubiquitin linkage, is not required for the ubiquitination and degradation of the LDLR.

The K48-linked polyubiquitin chain was first characterized as a proteasome delivery signal for short-lived cytosolic proteins (11, 12). In our previous study, we demonstrated that the autodegradation of the E3 ubiquitin ligase IDOL was mediated by the proteasomal pathway (29). It was therefore surprising to us that K48-specific ubiquitination of IDOL is not required for its degradation. A possible explanation for this discovery could be that the other ubiquitin linkages than K48 have partially redundant functions in proteasome-mediated protein degradation. In fact, it has been reported that other ubiquitin linkages can also be recognized by the proteasome (36). During mitosis, K11-linked ubiquitin chains bind proteasomal receptors and induce the degradation of cell cycle regulators (37–39). It is interesting to note that K63-specific ubiquitination has been reported to target proteasomal degradation of several proteins including Sic1, dihydrofolate reductase, cyclin B1, and troponin 1 (40–43), and therefore can serve as a competent proteolytic signal. In addition, the inhibition of the proteasome by MG132 in mammalian cells causes an increase of both K48- and K63-linked ubiquitin chains (43), suggesting a functional role of K63-linked ubiquitin in proteasomal degradation. Our results clearly indicate that when K48-specific ubiquitination is inhibited, it is possible for IDOL to use other ubiquitin linkages to carry out autoubiquitination and proteasome-dependent autodegradation.

In contrast to degradation of IDOL itself, the degradation of its primary biological target, the LDLR, occurs in the lysosome. Lysosomal sorting of several membrane proteins for degradation has been shown to be dependent on K63-specific ubiquitin linkage in yeast strains lacking endogenous ubiquitin (18–20). Although multiple recent studies in mammalian cells have also suggested an important role of K63-linked ubiquitin in the endocytosis and the lysosome sorting of membrane proteins (21–25), it is still not clear whether the K63-specific ubiquitination is indispensible for the lysosomal degradation of membrane proteins in a mammalian setting, because it has previously been difficult to effectively eliminate endogenous ubiquitin in mammalian cells. In this study, we have shown that the K63-specific ubiquitin linkage is not required for the ubiquitination and lysosomal degradation of the LDLR in mammalian cells. It is important to point out that although K63-linked ubiquitin chains appear to facilitate the degradation of membrane proteins, these proteins are not exclusively ubiquitinated via the K63-linkage. For example, interferon-α/β receptor 1 is polyubiquitinated via both K48- and K63-linked ubiquitin chains (44). Similarly, human epidermal growth factor receptor 2, a member in the EGFR family, also possesses both K48- and K63-linked ubiquitin chains when endocytosed (45). The mechanism by which K63-specific ubiquitination facilitates endocytosis and lysosomal sorting is not completely clear, but it has been suggested that the ubiquitin conformation in K63-linked chains provides better binding to a set of adaptor proteins that have ubiquitin binding domain, and that these adaptor proteins could initiate clathrin-dependent and -independent endocytosis (46–48). In the case of the LDLR, it is not clear whether its endocytosis would require these adaptor proteins. In addition, due to the multiple lysine and cysteine residues within its cytoplasmic tail, it is possible that simultaneous non-K63-linked ubiquitination on these residues could already provide enough affinity for adaptor binding.

Given the importance of the LDLR pathway in the liver, it will be of interest in the future to generate hepatocyte K48 and K63 ubiquitin replacement cell lines. It would be informative to have results from additional ubiquitin replacement cell lines expressing a double K48R/K63R ubiquitin mutant. Unfortunately, the generation of this reagent is exceedingly challenging. The only way to introduce mutant ubiquitins into a null background is to knockdown all 4 ubiquitin genes while simultaneously introducing the expression of a new ubiquitin molecule to keep the cells alive. It is very unlikely that a double K48R/K63R ubiquitin mutant would be viable after induction of replacement. The K48 mutant cells begin to die 48 h after induction of replacement due to the critical role of K48 linkages in protein degradation pathways. Double mutant cells would almost certainly die even sooner, making them impossible to study.

Overall, the lack of dependence on K48- or K63-specific ubiquitination in the degradation of IDOL and the LDLR is consistent with our previous finding that UBE2D is the cognate E2 in the ubiquitination of IDOL and the LDLR. UBE2D has been demonstrated to catalyze both K48- and K63-linked ubiquitin chains (41). In this context, it is not surprising that UBE2N/V1 is not required for the degradation of the LDLR, because UBE2N/V1 specifically catalyzes K63-specific ubiquitin linkage (32, 33). Although a previous report suggested a possible role for UBE2N/V1 in IDOL action, in our study we observed comparatively weak activity of this heterodimer on IDOL-dependent ubiquitination of either IDOL or the LDLR in vitro. We have also provided evidence in cultured cells that inactivation of UBE2N/V1 by either RNAi or dominant negative protein did not prevent the degradation of the LDLR. Therefore, UBE2N/V1 does not appear to be a major cognate E2 for the ubiquitination and degradation of the LDLR.

In summary, using the ubiquitin replacement strategy, we have systematically examined the requirement of K48- and K63-specific ubiquitin linkages in the ubiquitination and degradation of IDOL and the LDLR. Although we have demonstrated that these lysine-specific linkages are dispensable for both degradation pathways, the detailed function of ubiquitination in the degradation of IDOL and the LDLR remains to be elucidated in future studies.

## References

[1] Hershko A. Ciechanover A. 1998 The ubiquitin system. Annu. Rev. Biochem. 67: 425–479 9759494 10.1146/annurev.biochem.67.1.425

[2] Dye B. T. Schulman B. A. 2007 Structural mechanisms underlying posttranslational modification by ubiquitin-like proteins. Annu. Rev. Biophys. Biomol. Struct. 36: 131–150 17477837 10.1146/annurev.biophys.36.040306.132820

[3] Pickart C. M. 2001 Mechanisms underlying ubiquitination. Annu. Rev. Biochem. 70: 503–533 11395416 10.1146/annurev.biochem.70.1.503

[4] Komander D. Rape M. 2012 The ubiquitin code. Annu. Rev. Biochem. 81: 203–229 22524316 10.1146/annurev-biochem-060310-170328

[5] Dammer E. B. Na C. H. Xu P. Seyfried N. T. Duong D. M. Cheng D. Gearing M. Rees H. Lah J. J. Levey A. I. 2011 Polyubiquitin linkage profiles in three models of proteolytic stress suggest the etiology of Alzheimer disease. J. Biol. Chem. 286: 10457–10465 21278249 10.1074/jbc.M110.149633 PMC3060499

[6] Datta A. B. Hura G. L. Wolberger C. 2009 The structure and conformation of Lys63-linked tetraubiquitin. J. Mol. Biol. 392: 1117–1124 19664638 10.1016/j.jmb.2009.07.090 PMC2762427

[7] Eddins M. J. Varadan R. Fushman D. Pickart C. M. Wolberger C. 2007 Crystal structure and solution NMR studies of Lys48-linked tetraubiquitin at neutral pH. J. Mol. Biol. 367: 204–211 17240395 10.1016/j.jmb.2006.12.065

[8] Boname J. M. Thomas M. Stagg H. R. Xu P. Peng J. Lehner P. J. 2010 Efficient internalization of MHC I requires lysine-11 and lysine-63 mixed linkage polyubiquitin chains. Traffic. 11: 210–220 19948006 10.1111/j.1600-0854.2009.01011.x PMC3551259

[9] Dynek J. N. Goncharov T. Dueber E. C. Fedorova A. V. Izrael-Tomasevic A. Phu L. Helgason E. Fairbrother W. J. Deshayes K. Kirkpatrick D. S. 2010 c-IAP1 and UbcH5 promote K11-linked polyubiquitination of RIP1 in TNF signalling. EMBO J. 29: 4198–4209 21113135 10.1038/emboj.2010.300 PMC3018797

[10] Gerlach B. Cordier S. M. Schmukle A. C. Emmerich C. H. Rieser E. Haas T. L. Webb A. I. Rickard J. A. Anderton H. Wong W. W. 2011 Linear ubiquitination prevents inflammation and regulates immune signalling. Nature. 471: 591–596 21455173 10.1038/nature09816

[11] Chau V. Tobias J. W. Bachmair A. Marriott D. Ecker D. J. Gonda D. K. Varshavsky A. 1989 A multiubiquitin chain is confined to specific lysine in a targeted short-lived protein. Science. 243: 1576–1583 2538923 10.1126/science.2538923

[12] Finley D. Sadis S. Monia B. P. Boucher P. Ecker D. J. Crooke S. T. Chau V. 1994 Inhibition of proteolysis and cell cycle progression in a multiubiquitination-deficient yeast mutant. Mol. Cell. Biol. 14: 5501–5509 8035826 10.1128/mcb.14.8.5501-5509.1994 PMC359070

[13] Spence J. Sadis S. Haas A. L. Finley D. 1995 A ubiquitin mutant with specific defects in DNA repair and multiubiquitination. Mol. Cell. Biol. 15: 1265–1273 7862120 10.1128/mcb.15.3.1265 PMC230349

[14] Arnason T. Ellison M. J. 1994 Stress resistance in Saccharomyces cerevisiae is strongly correlated with assembly of a novel type of multiubiquitin chain. Mol. Cell. Biol. 14: 7876–7883 7969127 10.1128/mcb.14.12.7876-7883.1994 PMC359326

[15] Sun L. Deng L. Ea C. K. Xia Z. P. Chen Z. J. 2004 The TRAF6 ubiquitin ligase and TAK1 kinase mediate IKK activation by BCL10 and MALT1 in T lymphocytes. Mol. Cell. 14: 289–301 15125833 10.1016/s1097-2765(04)00236-9

[16] Mukhopadhyay D. Riezman H. 2007 Proteasome-independent functions of ubiquitin in endocytosis and signaling. Science. 315: 201–205 17218518 10.1126/science.1127085

[17] Papa F. R. Hochstrasser M. 1993 The yeast DOA4 gene encodes a deubiquitinating enzyme related to a product of the human tre-2 oncogene. Nature. 366: 313–319 8247125 10.1038/366313a0

[18] Galan J. M. Haguenauer-Tsapis R. 1997 Ubiquitin lys63 is involved in ubiquitination of a yeast plasma membrane protein. EMBO J. 16: 5847–5854 9312043 10.1093/emboj/16.19.5847 PMC1170216

[19] Springael J. Y. Galan J. M. Haguenauer-Tsapis R. Andre B. 1999 NH4+-induced down-regulation of the Saccharomyces cerevisiae Gap1p permease involves its ubiquitination with lysine-63-linked chains. J. Cell Sci. 112: 1375–1383 10194416 10.1242/jcs.112.9.1375

[20] Lauwers E. Jacob C. Andre B. 2009 K63-linked ubiquitin chains as a specific signal for protein sorting into the multivesicular body pathway. J. Cell Biol. 185: 493–502 19398763 10.1083/jcb.200810114 PMC2700384

[21] Huang F. Kirkpatrick D. Jiang X. Gygi S. Sorkin A. 2006 Differential regulation of EGF receptor internalization and degradation by multiubiquitination within the kinase domain. Mol. Cell. 21: 737–748 16543144 10.1016/j.molcel.2006.02.018

[22] Stang E. Blystad F. D. Kazazic M. Bertelsen V. Brodahl T. Raiborg C. Stenmark H. Madshus I. H. 2004 Cbl-dependent ubiquitination is required for progression of EGF receptors into clathrin-coated pits. Mol. Biol. Cell. 15: 3591–3604 15194809 10.1091/mbc.E04-01-0041 PMC491821

[23] Geetha T. Jiang J. Wooten M. W. 2005 Lysine 63 polyubiquitination of the nerve growth factor receptor TrkA directs internalization and signaling. Mol. Cell. 20: 301–312 16246731 10.1016/j.molcel.2005.09.014

[24] Duncan L. M. Piper S. Dodd R. B. Saville M. K. Sanderson C. M. Luzio J. P. Lehner P. J. 2006 Lysine-63-linked ubiquitination is required for endolysosomal degradation of class I molecules. EMBO J. 25: 1635–1645 16601694 10.1038/sj.emboj.7601056 PMC1440841

[25] Varghese B. Barriere H. Carbone C. J. Banerjee A. Swaminathan G. Plotnikov A. Xu P. Peng J. Goffin V. Lukacs G. L. 2008 Polyubiquitination of prolactin receptor stimulates its internalization, postinternalization sorting, and degradation via the lysosomal pathway. Mol. Cell. Biol. 28: 5275–5287 18573876 10.1128/MCB.00350-08 PMC2519723

[26] Hong C. Duit S. Jalonen P. Out R. Scheer L. Sorrentino V. Boyadjian R. Rodenburg K. W. Foley E. Korhonen L. 2010 The E3 ubiquitin ligase IDOL induces the degradation of the low density lipoprotein receptor family members VLDLR and ApoER2. J. Biol. Chem. 285: 19720–19726 20427281 10.1074/jbc.M110.123729 PMC2888382

[27] Scotti E. Hong C. Yoshinaga Y. Tu Y. Hu Y. Zelcer N. Boyadjian R. de Jong P. J. Young S. G. Fong L. G. 2011 Targeted disruption of the idol gene alters cellular regulation of the low-density lipoprotein receptor by sterols and liver x receptor agonists. Mol. Cell. Biol. 31: 1885–1893 21343340 10.1128/MCB.01469-10 PMC3133228

[28] Zelcer N. Hong C. Boyadjian R. Tontonoz P. 2009 LXR regulates cholesterol uptake through Idol-dependent ubiquitination of the LDL receptor. Science. 325: 100–104 19520913 10.1126/science.1168974 PMC2777523

[29] Zhang L. Fairall L. Goult B. T. Calkin A. C. Hong C. Millard C. J. Tontonoz P. Schwabe J. W. 2011 The IDOL-UBE2D complex mediates sterol-dependent degradation of the LDL receptor. Genes Dev. 25: 1262–1274 21685362 10.1101/gad.2056211 PMC3127428

[30] Sorrentino V. Scheer L. Santos A. Reits E. Bleijlevens B. Zelcer N. 2011 Distinct functional domains contribute to degradation of the low density lipoprotein receptor (LDLR) by the E3 ubiquitin ligase inducible degrader of the LDLR (IDOL). J. Biol. Chem. 286: 30190–30199 21734303 10.1074/jbc.M111.249557 PMC3191058

[31] Xu M. Skaug B. Zeng W. Chen Z. J. 2009 A ubiquitin replacement strategy in human cells reveals distinct mechanisms of IKK activation by TNFalpha and IL-1beta. Mol. Cell. 36: 302–314 19854138 10.1016/j.molcel.2009.10.002 PMC2779160

[32] Hofmann R. M. Pickart C. M. 1999 Noncanonical MMS2-encoded ubiquitin-conjugating enzyme functions in assembly of novel polyubiquitin chains for DNA repair. Cell. 96: 645–653 10089880 10.1016/s0092-8674(00)80575-9

[33] VanDemark A. P. Hofmann R. M. Tsui C. Pickart C. M. Wolberger C. 2001 Molecular insights into polyubiquitin chain assembly: crystal structure of the Mms2/Ubc13 heterodimer. Cell. 105: 711–720 11440714 10.1016/s0092-8674(01)00387-7

[34] Song B. L. DeBose-Boyd R. A. 2004 Ubiquitination of 3-hydroxy-3-methylglutaryl-CoA reductase in permeabilized cells mediated by cytosolic E1 and a putative membrane-bound ubiquitin ligase. J. Biol. Chem. 279: 28798–28806 15090540 10.1074/jbc.M402442200

[35] Gonen H. Bercovich B. Orian A. Carrano A. Takizawa C. Yamanaka K. Pagano M. Iwai K. Ciechanover A. 1999 Identification of the ubiquitin carrier proteins, E2s, involved in signal-induced conjugation and subsequent degradation of IkappaBalpha. J. Biol. Chem. 274: 14823–14830 10329681 10.1074/jbc.274.21.14823

[36] Thrower J. S. Hoffman L. Rechsteiner M. Pickart C. M. 2000 Recognition of the polyubiquitin proteolytic signal. EMBO J. 19: 94–102 10619848 10.1093/emboj/19.1.94 PMC1171781

[37] Jin L. Williamson A. Banerjee S. Philipp I. Rape M. 2008 Mechanism of ubiquitin-chain formation by the human anaphase-promoting complex. Cell. 133: 653–665 18485873 10.1016/j.cell.2008.04.012 PMC2696189

[38] Matsumoto M. L. Wickliffe K. E. Dong K. C. Yu C. Bosanac I. Bustos D. Phu L. Kirkpatrick D. S. Hymowitz S. G. Rape M. 2010 K11-linked polyubiquitination in cell cycle control revealed by a K11 linkage-specific antibody. Mol. Cell. 39: 477–484 20655260 10.1016/j.molcel.2010.07.001

[39] Williamson A. Wickliffe K. E. Mellone B. G. Song L. Karpen G. H. Rape M. 2009 Identification of a physiological E2 module for the human anaphase-promoting complex. Proc. Natl. Acad. Sci. USA. 106: 18213–18218 19822757 10.1073/pnas.0907887106 PMC2775311

[40] Hofmann R. M. Pickart C. M. 2001 In vitro assembly and recognition of Lys-63 polyubiquitin chains. J. Biol. Chem. 276: 27936–27943 11369780 10.1074/jbc.M103378200

[41] Kim H. T. Kim K. P. Lledias F. Kisselev A. F. Scaglione K. M. Skowyra D. Gygi S. P. Goldberg A. L. 2007 Certain pairs of ubiquitin-conjugating enzymes (E2s) and ubiquitin-protein ligases (E3s) synthesize nondegradable forked ubiquitin chains containing all possible isopeptide linkages. J. Biol. Chem. 282: 17375–17386 17426036 10.1074/jbc.M609659200

[42] Kirkpatrick D. S. Hathaway N. A. Hanna J. Elsasser S. Rush J. Finley D. King R. W. Gygi S. P. 2006 Quantitative analysis of in vitro ubiquitinated cyclin B1 reveals complex chain topology. Nat. Cell Biol. 8: 700–710 16799550 10.1038/ncb1436

[43] Saeki Y. Kudo T. Sone T. Kikuchi Y. Yokosawa H. Toh-e A. Tanaka K. 2009 Lysine 63-linked polyubiquitin chain may serve as a targeting signal for the 26S proteasome. EMBO J. 28: 359–371 19153599 10.1038/emboj.2008.305 PMC2646160

[44] Kumar K. G. Barriere H. Carbone C. J. Liu J. Swaminathan G. Xu P. Li Y. Baker D. P. Peng J. Lukacs G. L. 2007 Site-specific ubiquitination exposes a linear motif to promote interferon-alpha receptor endocytosis. J. Cell Biol. 179: 935–950 18056411 10.1083/jcb.200706034 PMC2099190

[45] Marx C. Held J. M. Gibson B. W. Benz C. C. 2010 ErbB2 trafficking and degradation associated with K48 and K63 polyubiquitination. Cancer Res. 70: 3709–3717 20406983 10.1158/0008-5472.CAN-09-3768 PMC2862137

[46] Chen H. De Camilli P. 2005 The association of epsin with ubiquitinated cargo along the endocytic pathway is negatively regulated by its interaction with clathrin. Proc. Natl. Acad. Sci. USA. 102: 2766–2771 15701696 10.1073/pnas.0409719102 PMC549477

[47] Sigismund S. Woelk T. Puri C. Maspero E. Tacchetti C. Transidico P. Di Fiore P. P. Polo S. 2005 Clathrin-independent endocytosis of ubiquitinated cargos. Proc. Natl. Acad. Sci. USA. 102: 2760–2765 15701692 10.1073/pnas.0409817102 PMC549482

[48] Varadan R. Assfalg M. Haririnia A. Raasi S. Pickart C. Fushman D. 2004 Solution conformation of Lys63-linked di-ubiquitin chain provides clues to functional diversity of polyubiquitin signaling. J. Biol. Chem. 279: 7055–7063 14645257 10.1074/jbc.M309184200

